# Analysis and Optimization of Spiral Circular Inductive Coupling Link for Bio-Implanted Applications on Air and within Human Tissue

**DOI:** 10.3390/s140711522

**Published:** 2014-06-30

**Authors:** Saad Mutashar, Mahammad A. Hannan, Salina A. Samad, Aini Hussain

**Affiliations:** 1 Department of Electrical, Electronic & Systems Engineering, Faculty of Engineering and Built Environment, Universiti Kebangsaan Malaysia, 43600 UKM Bangi Selangor, Malaysia; E-Mails: hannan@eng.ukm.my (M.A.H.); salina@eng.ukm.my (S.A.S.); aini@eng.ukm.my (A.H.); 2 Department of Electrical and Electronic Engineering, University of Technology-Iraq, 35010 Baghdad, Iraq

**Keywords:** bio-medical implanted devices, ISM band, inductive coupling link spiral coils, SAR effect

## Abstract

The use of wireless communication using inductive links to transfer data and power to implantable microsystems to stimulate and monitor nerves and muscles is increasing. This paper deals with the development of the theoretical analysis and optimization of an inductive link based on coupling and on spiral circular coil geometry. The coil dimensions offer 22 mm of mutual distance in air. However, at 6 mm of distance, the coils offer a power transmission efficiency of 80% in the optimum case and 73% in the worst case via low input impedance, whereas, transmission efficiency is 45% and 32%, respectively, via high input impedance. The simulations were performed in air and with two types of simulated human biological tissues such as dry and wet-skin using a depth of 6 mm. The performance results expound that the combined magnitude of the electric field components surrounding the external coil is approximately 98% of that in air, and for an internal coil, it is approximately 50%, respectively. It can be seen that the gain surrounding coils is almost constant and confirms the omnidirectional pattern associated with such loop antennas which reduces the effect of non-alignment between the two coils. The results also show that the specific absorption rate (SAR) and power loss within the tissue are lower than that of the standard level. Thus, the tissue will not be damaged anymore.

## 1. Introduction

The reduction of patient discomfort and risk of infection is the main design concern in the implanted device field. Recently, most bio-implantable devices are powered using implanted batteries, resulting skin infections and hazards. Because the implanted batteries have chemical side effects and limited lifetime, researchers have developed a safer alternative method for powering implantable devices using inductive power links. The magnetic inductive link approach is believed to be the most promising technique for implantable devices. Its advantages include continuous availability of high levels of power to an implanted device, and the ability to control it with the external device using the magnetic link. In addition, the lifetime and shelf life of the implanted devices are not restricted by the lifetime of a battery. Hence, it can be used for long term and during the patient's activities [1]

Bio-implantable devices designed with the smallest possible size are to be implanted depending on functional depth in the human biological tissue, which is usually less than 10 mm of depth. For example, in general implanted micro-system stimulators depth is 1–4 mm, for retinal implants it is 5 mm and for cochlear implants depth is 3–6 mm, respectively [2–4]. The inductive powering links method is a suitable method for powering implanted devices. This inductive coupling link consists of two *RLC* circuits, acting as a transmitter and receiver coils. To obtain better power transfer efficiency (PTE), both *RLC* circuits are tuned to the same resonant frequency ω_o_. The external *RLC* circuit fixed outside the body is tuned to the serial resonance to provide a low-impedance load to deliver maximum power from the source to the implanted circuit. The internal *RLC* circuit is implanted within the body and tuned to the parallel resonance to improve the driving performance for remote load resistance [5–7]. Many design issues should be considered such as patient comfort, endurance portability, reliability, size, power consumption and feasibility.

Most implanted microsystems are used to stimulate and monitor biological signals in the near field region such as nerves and muscle activities. In addition, the development of implanted microsystems will helps biological research to recognize and develop treatments for nerve diseases such as brain disorders *i.e.*, epilepsy, stroke and paralysis. Power transmission is the key in designing such bio-implantable devices. The power transfer efficiency is affected by the coil parameters, including their lumped elements such as parasitic resistance and capacitance associated with them, geometries, alignments, and distance between coils.

The mutual distance between the transmitter (external) and receiver (implant) coils is to be smaller than the wavelength in the near field, which depends on the coil dimensions. Hence, the coil dimensions have a direct effect on the distance and a coupling link which consists of two differing or same-sized coils, oriented face-to-face. The external coil located outside the body can be accurate with flexibility in the design in terms of size, while the implanted coil located within the human body should be as small as possible. The implanted coil is the main reason for the power consumption, therefore; it should be carefully designed in terms of shape and geometry. The coupling increases as the coil dimensions increase and as the distance between the coils decreases. To provide a coupling link at 10 mm, a spiral rectangular coil shape with external and internal dimensions of 62 mm × 25 mm and 25 mm × 10 mm was designed at 13.56 MHz. In this design, the relative short-range coupling and implanted coil size are the issues [8].

A spiral square coil shape with outer and inner dimensions of 70 mm × 8 mm and 20 mm × 8 mm was designed for 1 MHz to 5 MHz frequency ranges. It is seen that the design provides optimum coupling at a distance of 10 mm; however, for better performance, the size and relative short-range coupling still need to be considered [9]. The coupling links between two circular coil dimensions d_out_ = 38 mm, d_in_ = 36 mm and d_out_ = 18 mm, d_in_ = 16 mm was designed to offer 15 mm of distance coupling with an operating frequency of 742 KHz. However, this design will increase the printed board circuits and occupies a relatively large area within tissues [10]. For EEG signal detection, Sauer *et al.*, designed a coupling link based on circular coils with external coil dimensions of 50 mm and an internal coil of 20 mm to offer 28 mm of distance with an operating frequency of 4 MHz, the implanted coil size is still the issue when it occupies a relatively large area [11].

Printed spiral circular coils (PSCCs) are robust against lateral misalignments and reliable for implantation under the skin. They can be fitted outside the body or within the epidural space. In this study, the reflected impedance concept analysis is optimized and modified to obtain efficient power transmission between two pairs of proposed single-layer planar spiral circular coils, which are printed on small substrate PCBs. Firstly, air separates the two coils. Then, a biological human tissue model with 6 mm of thickness is assumed as a medium between coils, which is considered as a relative distance between the coils. The tissue thickness of 6 mm is an appropriate depth, which is faithful to the distance used for implantable devices such as cochlear implants, implanted micro-systems and retinal implants [4]. The transmitter and receiver had the following respective external and internal dimensions: the outer and inner transmitter dimensions are 56 mm and 10 mm, respectively, and for the receiver (implanted) coil they are 11.6 mm and 5 mm, respectively. The circuit was designed to provide a 22 mm of coupling distance using the Industrial Specific Medical (ISM) operating frequency of 13.56 MHz. The distance of 22 mm is more than enough for our application because the proposed tissue thickness is no more than 6 mm. The geometry of the coil values is recommended by theoretical calculations using MATLAB to establish the efficiency of the coils. The High Frequency Structure Simulator ANSOFT-HFSS-13.0 with finite element method (FEM) software in near-field is used to simulate and calculate the gain surrounding coils in air and within the tissue; it is also used to validate the coil design. The SEMCAD software used to calculate the SAR within tissue.

## 2. Methods and Materials

An inductive coupling link with serial to parallel topology is a suitable connection for biomedical applications. The theoretical analysis and simulation are very important in designing an ideal inductive coupling for estimating the received power. Different theoretical analysis methods have been investigated for solving and deriving the equations for calculating the received power of the implant device.

In this paper, the reflected impedance method is obtained by modifying the lumped equivalent circuit to quantify and optimize the power transmission efficiency between the two coils. The optimization is done when the input impedance is low and when the input impedance is high. The medium separating the coils is considered as air (free space) and biological human tissue separately. The simulated biological human tissue model included dry and wet skin having similar constitutive parameters to live human tissue.

## 3. Analysis and Optimization Based on Coupling

There are four different combinations of highly coupled stagger-tuned inductive links. They are: voltage in-voltage out, voltage in-current out, current in-current out and current in-voltage out. The “voltage in-voltage out” link is an approach which can control the output power. This approach is based on the coupling used to calculate and optimize the power transmission efficiency.

### 3.1. Inductive Power Transmission Efficiency

**Figure 1.**
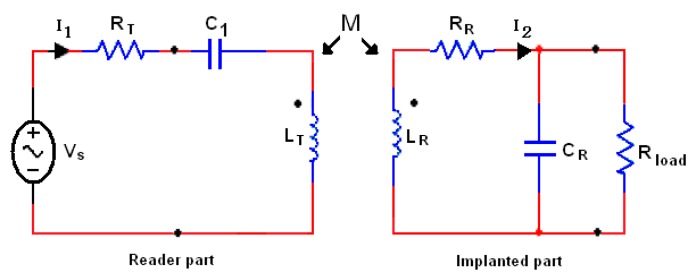
The inductive SP topology.

The inductive link with serial to parallel (SP) topology is the common part between the external and internal architectures which is the suitable topology to be used for subcutaneous implanted devices. Figure 1 shows the equivalent scheme of the SP topology that consists of two parts, external part (reader) and internal part (implant). The power transfer efficiency (PTE) is one of the major specifications for assessing the performance of an inductive link [12].

**Figure 2.**
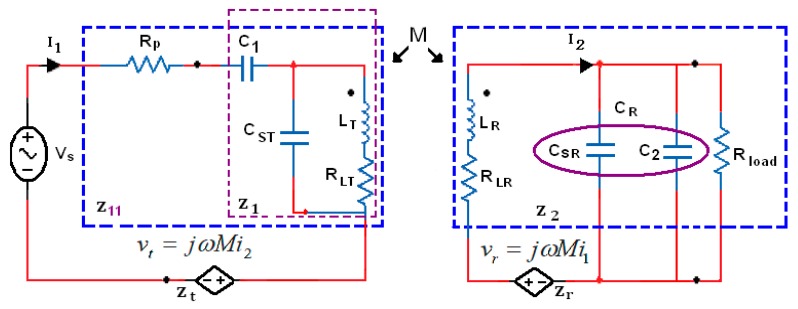
Inductive power link schematic and its equivalent circuit.

Figure 2 shows the lumped elements of the equivalent scheme, the resistance *R_LT_* and *R_LR_* are equivalent series resistors of inductors *L_T_* and *L_R_*, respectively, and expressed as follows:

(1) $$L_{T}=\frac{1}{C_{1}\omega_{0}^{2}}$$

(2) $$L_{R}=\frac{1}{C_{2}\omega_{0}^{2}}$$

(3) $$R_{LR}=\frac{R_{\text{load}}}{1+\omega_{0}^{2}R_{\text{load}}^{2}C_{R}^{2}}$$

The parasitic resistor *R_LT_* is model the series resistance of one turn of the inductance. This resistor also included the reflected back resistor into the transmitter which is very small and can be ignored. *M* is the mutual inductance between the transmitter inductance *L_T_* and receiver inductance *L_R_*. The primary coil driven by the efficient power amplifier, which is modeled as a voltage source and having a series resistance R_P_.

The coil windings have umped elements, such as parasitic resistance and capacitance associated with them. The transmitter and receiver coil lumped elements are R_LT_, C_ST_ and R_LR_, C_SR_, respectively. The capacitors C_1_ and C_2_ are added to form the resonant LC-tank circuits for the conductors L_T_ and L_R_, respectively, and should be chosen to approximately have the same values. R_load_ represents the load resistance, and for the simplest analysis, C_R_ = C_SR_ + C_2_. The efficient power transmission is calculated by using reflected impedance concept analysis from the receiver and transmitter, (Z_t_ and Z_r_) as given in Equations (4)–(7):

(4) $$Z_{t}=\frac{v_{t}}{I_{1}}=\frac{\omega^{2}M^{2}}{Z_{2}}$$

(5) $$Z_{r}=\frac{v_{r}}{I_{2}}=j\omega M\frac{I_{1}}{I_{2}}$$

(6) $$Z_{1}=(R_{LT}+j\omega L_{T}//\frac{1}{j\omega C_{ST}})+\frac{1}{j\omega C_{1}}$$

(7) $$Z_{2}=R_{LR}+j\omega L_{R}+\frac{1}{j\omega C_{R}}//R_{\text{load}}$$

where Z_1_ and Z_2_ represent the impedance network for the transmitter and receiver including real and imaginary parts. The power transmitter efficiency (defined as the ratio of power consumed by the secondary circuits over the total power drained from the power supply source η_T_) and the power receiver efficiency (defined as the ratio of power consumed by the load R_load_ over the total power consumed at the implanted side η_R_) as given in Equations (8) and (9), respectively:

(8) $$\eta_{T}=\left|\frac{Z_{t}}{Z_{1}+Z_{t}}\right|≦\frac{K^{2}Q_{1}Q_{2}R_{\text{load}}}{K^{2}Q_{1}Q_{2}R_{\text{load}}+Q_{2}^{2}R_{LR}}$$

(9) $$\eta_{R}=\left|1-\frac{R_{LR}}{Z_{2}}\right|≦\frac{Q_{2}^{2}R_{LR}}{Q_{2}^{2}R_{LR}+R_{\text{load}}}$$

Then total link efficiency is *η_link_* = *η_T_* * *η_R_*, where total link efficiency *η_link_* reaches its maximum if Z_1_ and Z_2_ has only the real part left where the imaginary part is approximately zero; Q_1_ and Q_2_ represent the quality factors of transmitter and receiver coils as follows:

(10) $$Q_{1}=\frac{\omega_{0}L_{T}}{R_{LT}}$$

(11) $$Q_{2}=\frac{\omega_{0}L_{R}}{R_{LR}}$$

Both coils tuned at the same resonant frequency as given in Equation (12):

(12) $$\omega =\omega_{0}=\frac{1}{{\left(L_{T}C_{1}\right)}^{\frac{1}{2}}}=\frac{1}{{\left(L_{R}C_{R}\right)}^{\frac{1}{2}}}$$

The coupling coefficient value is (0 < K < 1) depends on the coils' radius and is calculated as given in the coil optimization section. The implanted load resistance *R_load_*, is calculated according to [13], as expressed in Equation (13):

(13) $$R_{{\text{load}}^{2}}-4\omega^{2}L_{R}^{2}>0\;\text{where}\;\omega =2\pi \text{f}\;\text{and}\;{\text{R}}_{\text{load}}\geq 2\omega {\text{L}}_{\text{R}}$$

where hence the R_load_ ≥ 170 Ω, in our calculation we assume the R_load_ values are 200 Ω to 400 Ω by 50 Ω steps, where 200 Ω presents the worst case and 400 Ω is an optimum case. The total efficiency is a product of η*_T_* and η*_R_* as illustrated in Equation (14):

(14) $$\eta_{\text{total}}=\eta_{T}\eta_{R}=\frac{K^{2}Q_{1}Q_{2}^{3}R_{LR}R_{\text{load}}}{\left(K^{2}Q_{1}Q_{2}^{3}R_{LR}R_{\text{load}}+K^{2}Q_{1}Q_{2}R_{\text{load}}^{2}+Q_{2}^{4}R_{LR}^{2}+2Q_{2}^{2}R_{LR}R_{\text{load}}+R_{\text{load}}^{2}\right)}$$

The efficiency given in Equation (14) is perfect and appropriate in the absence of resistance of the power amplifier, source and the low external circuit impedance. The inductive coupling circuit is driven with an efficient subcutaneous power amplifier and this makes the external impedance increase and reduces the power delivered to the internal circuit. Therefore, the power amplifier should be carefully designed with optimum load resistance (R_P_) by using a matching network where the lumped elements for the external coil can also be determined [14]. The external impedance including the power amplifier resistance is as illustrated as follows:

(15) $$Z_{11}=[(R_{LT}+j\omega L_{T}//\frac{1}{j\omega C_{ST}})+\frac{1}{j\omega C_{1}}]+R_{P}$$

(16) $$\eta_{T}=\left|\frac{Z_{t}}{Z_{11}+Z_{t}}\right|$$

The PTE equation based on these factors given in the Figure 2 becomes:

(17) $$\eta_{\text{link}}=\frac{P_{O}}{P_{S}}=\frac{K^{2}Q_{1}Q_{2}^{3}R_{\text{load}}R_{LR}}{\left[K^{2}Q_{1}Q_{2}R_{\text{load}}+\left(1+\frac{R_{P}}{R_{LT}}\right)\left(R_{\text{load}}+Q_{2}^{2}R_{LR}\right)\right]\times \left(R_{\text{load}}+Q_{2}^{2}R_{LR}\right)}$$

Equations (14) and (17) are modeled in MATLAB based on the values given in the inductive link coil design section (Table 1). The power transfer efficiency for lower input impedance is higher than the efficiency with higher input impedance.

### 3.2. Optimization of Maximum Efficiency

The purpose of the theoretical analysis given above is to facilitate the process of the inductive coupling link optimization. Referring to Figure 2, the optimization is obtained for impedance Z_1_ and Z_2_ by reducing the implanted impedance Z_2_ without considering the power amplifier resistance. The power efficiency takes place at the resonant frequency ω_o_ on the implanted coil as follows:

(18) $$\omega_{o.\text{imp}}^{2}=\frac{1}{L_{R}C_{R}}-{\left(\frac{1}{C_{R}R_{\text{load}}}\right)}^{2}$$

From Equation (18), it is noted that the resonant frequency depends on L_R_, C_R_ and R_load_. Hence, if there is any change in the implanted load resistance, the coupling system will be out of resonance. Thus, the implanted remote will not receive the maximum transmitted power. The maximum transmitted power between the two coils is achieved when both *LC* circuits of the external and implanted coil have the same resonance frequency and the imaginary part of the impedance X_eq_ = 0 and this occurs when the load reflected in the transmitter has the same value as the impedance of the transmitter coil in resonance Z_1_ as follows:

(19) $$P_{\text{max}}=\frac{M^{2}}{L_{R}+\frac{R_{\text{load}}}{R_{\text{load}}C_{R}+1}+R_{LR}}$$

where *M* represents the mutual inductance between the external and internal coil, which is calculated in the optimization section. On the secondary side, a resonance capacitor *C_R_* cancels the inductive impedance of the receiving coil. The secondary resonance greatly enhances the performance of an inductive link, therefore, this capacitor playing a big rule in the efficiency enhancement. Referring to Equation (19), the optimization can be achieved by adjusting the capacitor *C_R_* to get optimum resonance as given in Equation (20):

(20) $$C_{R}=\frac{R_{L}\pm \sqrt{R_{L}^{2}-4L_{R}^{2}\omega_{0}^{2}}}{2L_{R}R_{\text{load}}\omega_{o}^{2}}$$

Hence, we optimize the power transmission efficiency of the inductive link, which is given in Equation (14), without considering the power amplifier resistance that can be expressed as given in Equation (21):

(21) $$\eta_{opt}=\frac{{\left|I_{2}\right|}^{2}R_{\text{load}}}{R_{eq}\left[I_{1}\right]V_{s}}$$

(22) $$R_{eq}=\frac{R_{LR}R_{\text{load}}\left(R_{\text{load}}+R_{LR}\right)+2\omega^{2}L_{\text{imp}}R_{\text{load}}}{{\left(R_{\text{load}}-\omega^{2}L_{\text{imp}}C_{R}R_{Lr}+R_{Lr}\right)}^{2}+{\left(2\omega L_{\text{imp}}+\omega C_{R}R_{Lr}R_{\text{load}}\right)}^{2}}$$

where R_eq_ presents the real part of the implant coil impedance and L_imp_ represents the implanted coil that involves lumped elements (L_R_, R_LR_, C_SR_).

## 4. Coils Design and Optimization

In general, the coil design and optimization can be achieved based on two methods−coupling based and coil geometry based. The optimization based on coupling is used to optimize the power transfer efficiency, data rate transmission, voltage gain and bandwidth. The optimization based on coil geometry is used to enhance the coil performance in air and within the tissue. In this section, the methodology to design the inductive link based on spiral circular coil geometry is introduced. This optimization can be achieved by developing rules and formulas presented in previous studies. The optimization is obtained based on the coil geometry parameters such as coil shape, outer and inner dimensions, mutual inductance (M), self-inductance (L), coefficient factor (K) and fill factor (φ).

### 4.1. Spiral Coil Characterization

**Figure 3.**
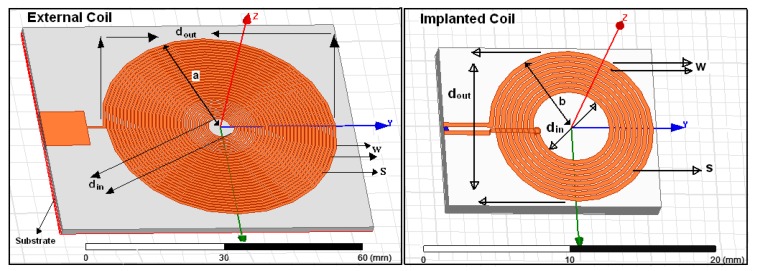
Coil geometries simulated by HFSS software.

The HFSS software is used to validate and simulate the proposed printed spiral circular coils and design the tissue model. In order to reduce the power losses in the transmission load, 1-0Z copper wire (30 AWG) with 0.036 mm thickness is printed on Rogers 4350™ substrate with constitutive parameters of 1.5 mm substrate thickness, 4.4 (FR4) dielectric constant, 3.66 relative permittivity (ε_r_), 0.004 dielectric loss tangent and “1” relative permeability (μ_r_), respectively. The implanted coil is coated by RH-5 substrate with ε_r_ = 1.0006 and loss tangent = 0. The external coil is printed on a substrate having dimensions of 70 × 60 × 1.5 mm while the internal coil is printed on a substrate having dimensions of 12 × 15 × 1.5 mm as shown in Figure 3. The calculated parameters of the transmitter and receiver coils will be explained in detail in the coil design section and as given in Table 1.

### 4.2. Optimization of Geometric Design Based on Circular Spiral Coils

Circular spiral coils are widely used in implanted devices as inductive coupling links due to their robustness against lateral coil misalignment. Because the external coil is fixed outside the body, it can be improved by increasing the dimensions of the coil. The design of the internal coil is limited by many factors such as shape, weight, number of turns and size, hence, the outer dimension of the implanted coil should be small, with a few turns, and should have a low consumption to prevent tissue heating. The proposed coils are used for the implanted microsystem in the radiating near-field (Fresnel) region at an operating frequency of 13.56 MHz in the arrow-band. The EM fields around the coils should be constant and confirm the omnidirectional pattern associated with such loop antennas, which allows better links with the implanted coil. In the case of circular loops, the distance of the radiation (D) is defined by the outer transmitted coil dimensions (*d_out_*) and should satisfy the condition adopted by Finkenzeller [15] as given in Equation (23):

(23) $$d_{\text{out}}\leq D2\sqrt{2}$$

The mutual distance between the transmitter and receiver circular coils should satisfy the expression Equation (24) [16] which we modified as given in Equation (25):

(24) $$a=\sqrt{X^{2}+b^{2}}$$

where a and b represents the external and implanted coils radius, respectively:

(25) $$\frac{d_{\text{out}.T}}{2}=\sqrt{X^{2}+{\left(\frac{d_{\text{out}.R}}{2}\right)}^{2}}$$

where *X* represents the mutual distance between coils and *d_out.T_*, *d_out.R_* presents the external and internal outer dimensions for the transmitter and receiver coil as shown in Figure 3.

The methods used in calculating the mutual inductance between the two coils and the self-inductance of such coils have been previously reported [17,18] and for the spiral circular coils they are adapted from [19]. The mutual inductance of two parallel filament coils (Δ = 0) and separated by X distance and having a d_T_ and d_R_ of dimensions can be expressed as follows:

(26) $$M(d_{T},d_{R},\Delta =0,X)=\frac{1}{2}\mu_{0}\sqrt{d_{T}\times d_{R}}\left[\left(\frac{2}{f}-f\right)K\left(f\right)-\frac{2}{f}E\left(f\right)\right]$$

where μ_0_ = 4π × 10^−9^ H/cm and represent the permeability of space, *K(f)*, *E(f)* represents the elliptic integrals given in Equation (27):

(27) $$f\left(d_{T},d_{R},\Delta =0,X\right)=\sqrt{\frac{4d_{T}d_{R}}{{\left(d_{T}+d_{R}\right)}^{2}+X^{2}}}$$

The summations of the partial mutual inductance for both coils need a complex analytical model. Hence, circular coils having a simple mathematical model are proposed, and the external and internal coils have a very small space between each turn as given in Table 1. Therefore, Lyle's method [20] is used for the considered external and internal coils having two circular filaments with dimensions *d_out.T_*, *d_in.T_* and *d_out.R_*, *d_in.R_*, respectively, and having an arithmetic geometric average as given in Equation (28):

(28) $$d_{\text{avg}}=0.5\times \left(d_{\text{out}}+d_{\text{in}}\right)$$

where *d_out_* and *d_in_* represent the outer and inner coil dimensions. Hence, the approximated mutual inductance between two spiral circular coils is found as a function of the number of turns for each coil, coil dimensions and distance, which can be developed and simplified as given in Equation (29) [15,21]:

(29) $$M≅\frac{\mu_{0}N_{T}d_{\text{out}.T}^{2}N_{R}d_{\text{out}.R}^{2}\pi }{2\sqrt{{\left(d_{\text{out}.R}^{2}+X^{2}\right)}^{3}}}$$

where *N_T_* and *N_R_* are the number of turns of the transmitter and receiver coils.

The self-inductance such as that of the spiral circular planar coil is determined based on the approximation of the spirals and equivalent current densities and be calculated as given in Equation (30):

(30) $$L=\frac{C_{1}\mu_{0}N^{2}d_{\text{avg}}}{2}\left[l_{n}\left(\frac{C_{2}}{\varphi }\right)+C_{3}\varphi +C_{4}\varphi^{2}\right]$$

where N represents the numbers of turns for such circular coil, C_i_ is a dependent coefficient, In this case it is a circle for which the coefficients are C_1_ = 1.00, C_2_ = 2.46, C_3_ = 0.00, C_4_ = 0.20 [18]. The arithmetic geometrical averages like the fill ratio defined as φ have to be computed and calculated as illustrated in Equation (31):

(31) $$\varphi =\frac{\left(d_{\text{out}}-d_{\text{in}}\right)}{\left(d_{\text{out}}+d_{\text{in}}\right)}$$

**Figure 4.**
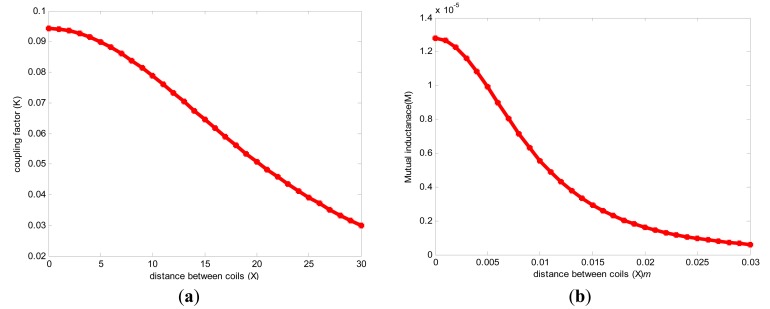
the relationship between (**a**) Coupling coefficient; (**b**) mutual inductance and distance between two coils.

To design and optimize our spiral circular coil (pancake) we used two concepts; the first one is based on Equation (24), where the external coils' radius is approximately equal to the distance to be covered (for a small internal coil). On the other hand, the mutual distance between the external and implanted coils is about twice the implanted coil diameter. The second one is based on the relations between the outer and internal coils. Then increasing mutual distance can be solved by optimizing the coil dimensions by making d_in.T_ ≈ 0.18 d_out.T_ and d_in.R_ ≈ 0.75 d_out.R_ respectively, [19]. Using the equation approximations above and the chart from Figure 4, the external coil outer dimensions are assumed d_out.T_ = 56 mm then d_in.T_ = 10 mm, the implanted coil dimensions found d_out.R_ = 11.6 mm, and d_in.R_ = 5 mm, respectively [22]. To validate our design optimization, the values above were inserted into Equation (25), giving as mutual distance result X = 22 mm which is much closer to the radiated distance from the external coil given in Equation (23) where D = 20 mm. The coefficient factor (*K*) is calculated based on coils' dimensions. Since its for an implanted microsystem usually d_out.T_ ≥ d_out.R_ and according to the values given in Table 1. In our applications we used a tissue thickness of 6 mm as a mutual distance between the coils, hence, the coefficient coupling (*K*) is calculated as follows:

(32) $$K=\frac{a^{2}xb^{2}}{\sqrt{ab}{\left(\sqrt{a^{2}+X^{2}}\right)}^{3}}$$

To validate the parameters *M* and *K*, Equations (29) and (32) were modulated in MATLAB as shown in Figure 4,b, respectively.

For information and clarification, the dielectric constitutive parameters of the biological human tissue at low band frequencies such as 13.56 MHz are not available in the specialized scientific research and literature; approximate body tissue dielectric data are available in [23].

## 5. Human Biological Tissue and SAR at 13.56 MHz

The implanted coil receives the energy transferred by external coils through the human tissue. Therefore, the human tissue must be taken into account. Because it is not allowed to use the living human tissue in testing, and cadavers are not suitable for testing because they lose the tissue properties 10 h after death, and their temperature becomes lower than 22°. In addition, the use of the animal tissue (e.g., ground beef, pig tissue and mouse tissue) does not give the real desired results because of the totally different constitutive parameters between the human and the animal tissue. As a solution, we designed the biological human tissue model with its constitutive parameters in HFSS and used it as a medium separating the two parts.

The conductivity, permeability and the loss-tangent of the human tissue are considered in our proposed design, while other designers did not consider such constitutive parameters of all types of the tissue layers in their designs or they only considered air as the main medium [9,24,25]. They claimed that the frequencies below 20 MHz couldn't damage the tissue, so most designers haven't considered the tissue when the operating frequency is lower than 20 MHz or they used only one type of tissue.

**Figure 5.**
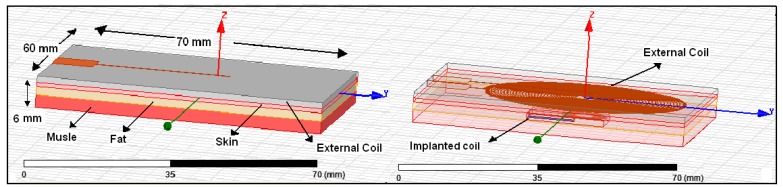
Pancake coils simulated with a proposed dimension of a human biological tissue model.

Therefore, in this work, two types of biological human tissue with wet and dry skin are proposed as the medium separating the coils. The external coil contacts the skin, and the internal coil is implanted within the tissue at a depth of 6 mm. Hence, the magnetic flux links the implanted coil and the external coil and transfers power to the implanted coil. Each type of proposed human biological tissue consists of three layers, the first layer is the skin with 1 mm thickness, the second layer is the fat with 2 mm thickness and the third layer is muscle with 3 mm thickness. The total tissue dimension is 70 mm × 60 mm × 6 mm and it is designed using HFSS as shown in Figure 5. In this process, the EMF is solved at 13.56 MHz.

The implanted and external coils surrounding gain was defined on a sphere of radius 12 cm and 28 cm, respectively, which defines the region around the antenna. Our chosen frequency is 13.56 MHz and is compared with the International Commission on Non-Ionizing Radiation Protection (ICNIRP) and Federal Communications Commission (FCC) standards. Both standards require that the SAR level be at or below 1.6 Wkg^−1^ spread over a volume of 1 g of tissue, whereas the SAR limit is 2 Wkg^−1^ averaged over 10 g of tissue [25]. At frequencies lower than 20 MHz, the SAR is very small compared with the SAR standard. Therefore, to validate that the proposed internal coil cannot damage the tissue due to electromagnetic absorption, the high accuracy field solver SEMCAD 14.6 was used to calculate the SAR using numerical techniques with a finite-difference time domain (FDTD).

## 6. SAR and Power Losses Calculation inside the Tissue

In order to estimate the power losses inside the human body, numerical calculation methods can be used. In general, determining SAR can be achieved by using numerical techniques or experimental methods by using fabricated tissue phantoms, where the given results are not close to reality, in addition the power losses cannot be determined within tissue [26]. Other studies have tried to fabricate gels exhibiting similar electrical properties as tissues for testing implantable devices and these gels are used to examine the interaction between electromagnetic waves and biological tissues. All this gel was fabricated according to the Industrial, Scientific and Medical (ISM) 2.4–2.48 GHz band, and according to the medical implant communications service (MICS) 402–405 MHz one [27]. The gels are fabricated by making an artificial material which shows electrical features approximately similar to human skin tissue. The contents of this material are oil, sugar, Triton, agarose, NaCl and deionized water). This fabricated gel suffered from several problems such as quick loss water as a result of the evaporation process and it cannot keep a fixed temperature like the temperature of the human body. Thus it loses its properties quickly, and hence it must be kept in designated places. For our work, we didn't find any studies that fabricated gels for low band frequency (1–20 MHz). In addition, we believe that whatever the accuracy of the tissue phantom manufacturer, it cannot be absolutely similar to the living human tissue.

As a solution, we used such specific and accurate software (SIMCAD) with a high resolution 3-D human body model, and we selected the position of the implanted coil and the tissue with its real constitutive parameters at 13.56 MHz [28]. Then, to calculate SAR and power losses within tissue, the implanted coil was designed using Pspice, which it is compatible with the SEMCAD software, and powered it with five voltages and 150 mW. Then the proposed implanted coil was inserted into the 3D model at the top of the adult human head in the space between the skull and brain at a depth of less than 1 cm. The SEMCAD software provides a high level of anatomical details and avoids using practical experiments on live human tissue. These models play an important role in optimizing the evaluation of electromagnetic exposures, e.g., in human body models [29]. A future task of this study will be to fabricate phantom tissues having constitutive parameters at 13.56 MHz according to [29] but this fabrication may only be done by certain companies.

## 7. Results and Discussion

In this study, a theoretical analysis of PS topology inductive coupling links is presented. The mathematical analysis is modeled in MATLAB using the reflected impedance method to determine the power transmission efficiency between the two parts. The reflected impedance concept analysis is optimized and modified to obtain efficient power transmission between the two pairs of proposed single-layer planar spiral circular coils, which are printed on small PCBs made of substrate. In the reflected impedance method, two approaches were used; the first approach is the analysis done without considering the power amplifier resistance, and the second approach is with power amplifier resistance. The implanted load resistor is considered 200 Ω to 400 Ω, and for implanted microsystem purposes, the needed distance between coils is 6 mm with a coefficient factor of 0.087 and mutual inductance of 0.95 μH.

**Figure 6.**
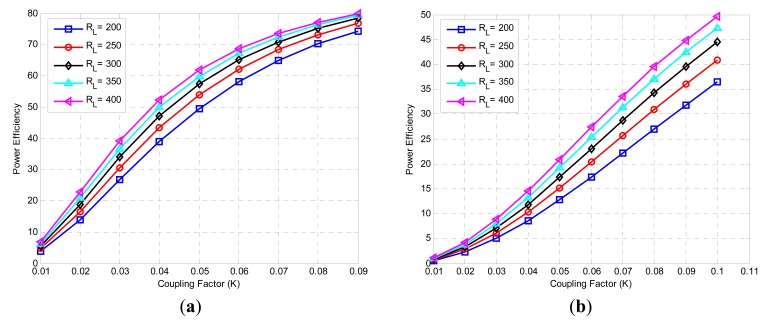
Power efficiency links (**a**) with low and high impedance; (**b**) with low input impedance and various load resistances.

Figure 6a,b shows the variation of power efficiency with various resistances and coefficient factors with high and low input impedance, respectively. The maximum efficiency without power amplifier resistor is 80%, and the worst efficiency is 73%, whereas, the maximum efficiency with the power amplifier consideration is 45% and the worst efficiency is 32%. The results showed that the power efficiency with power amplifier resistance (high input impedance) is approximately 55% more than that without power amplifier resistance (low input impedance).

To test the proposed coils' performance, and to find the natural fields surrounding the coil in air and in human biological tissue, the model is analyzed and optimized using the commercial field solver HFSS 13.0. The coils were investigated in two conditions; the first condition was when air (free space) separated the coils placed at distances of 6 mm. The second condition was when simulated human biological tissue with dry and wet skin replaced the air.

### 7.1. Near-Fields Surrounding Coils on Air

**Figure 7.**
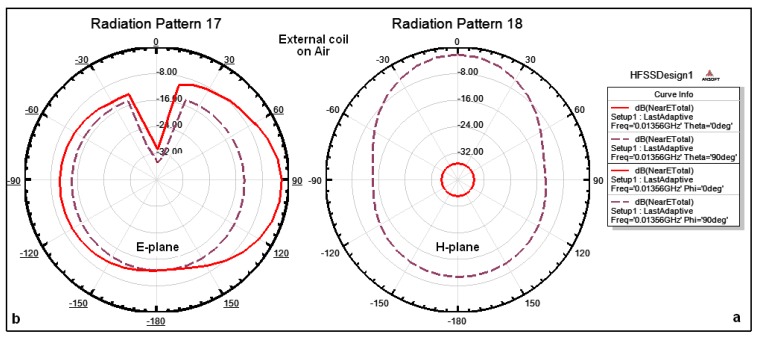
Simulated patterns for total near field surrounding external coil on air: (**a**) on the azimuthal plane (θ = 0°, θ = 90°); (**b**) on the elevation plane (Φ = 0°, Φ = 90°), respectively.

The combined magnitude of the electric field components (elevation and azimuthal planes) for external and internal coils in air can be explained as follows: Figure 7 illustrates the simulated natural E-Total near-field patterns surrounding external coil on air at a distance of 6 mm, where Figure 7a shows that the total near field on the azimuthal plane (θ = 0°, θ = 90°) are approximately −36 dB and –3 dB, respectively, and the surrounding fields are omnidirectional at all angles. Figure 7b shows that the surrounding fields on the elevation plane (Φ = 0°, Φ = 90°) are approximately −3 dB and −28 dB. The magnitude of the electric field surrounding the coil is omnidirectional and there is a drop and change in the magnitude in some specific angles.

**Figure 8.**
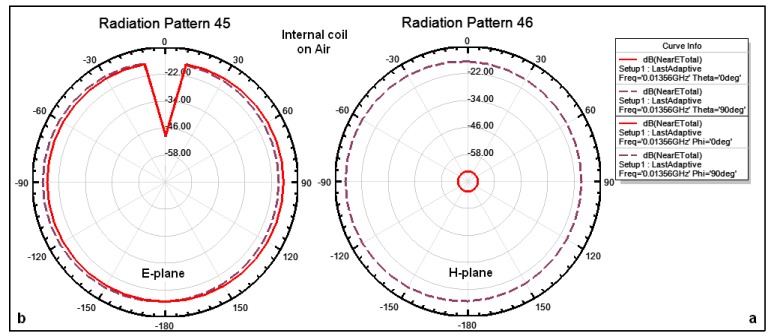
Simulated patterns for total near field surrounding an internal coil on air: (**a**) on the azimuthal plane (θ = 0°, θ = 90°); (**b**) on the elevation plane (Φ = 0°, Φ = 90°), respectively.

The outer dimensions of the implanted coil are smaller than those of the external coil; therefore the gain surrounding the implanted coil is lower than the gain surrounding the external coil. Figure 8 illustrates the simulated natural fields surrounding the internal coils in air at 6 mm distance where Figure 8a shows that the total near fields on the azimuthal plane (θ = 0°, θ = 90°) are approximately −68 dB and −18 dB, respectively, and the magnitude of the electric field surrounding the coil is omnidirectional at all angles. Figure 8b shows that the total near fields on the elevation plane for both Φ = 0°, Φ = 90° are approximately −22 dB. The magnitude of the electric field surrounding the coil is omnidirectional and there is a drop in the magnitude at some specific angles of up to −48 dB.

### 7.2. Near-Fields Surrounding Coils within Tissue

**Figure 9.**
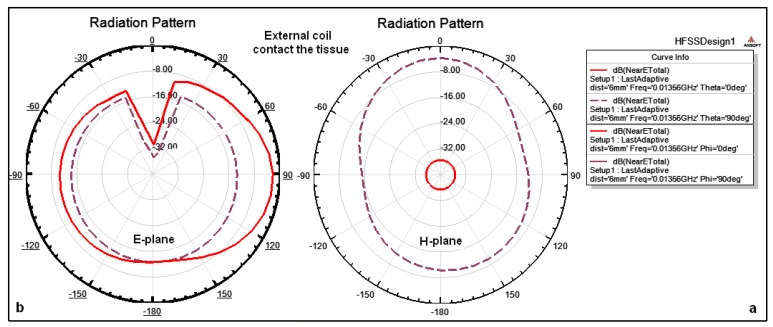
Simulated patterns for total near field surrounding external coil when it contacts the tissue: (**a**) On the azimuthal plane (θ = 0°, θ = 90°); (**b**) on the elevation plane (Φ = 0°, Φ = 90°), respectively.

The external coil contacts the skin and the internal coil is implanted inside the tissue at a depth of 6 mm. Therefore, the combined magnitude of the electric field components for external and internal coils within tissue can be explained as follows: Figure 9 illustrates the simulation of the near-field patterns E-Total surrounding the external coil on the azimuthal plane (θ = 0°, θ = 90°), and on the elevation plane (Φ = 0°, Φ = 90°). Because the external coil just contacts the tissue therefore, there is no tissue effect on the coil performance and the amount of the surrounding coil is similar to the results given in Figure 7.

**Figure 10.**
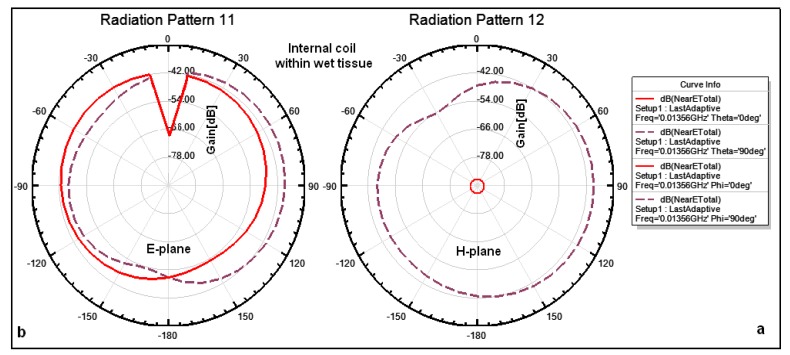
Simulated patterns for total near field surrounding an internal coil within tissue: (**a**) on the azimuthal plane (θ = 0°, θ = 90°); (**b**) on the elevation plane (Φ = 0°, Φ = 90°), respectively.

The internal coil is implanted in the tissue at a depth of 6 mm, hence the coil performance is different from the coil performance when it is in free space. Figure 10 illustrates the simulated natural fields surrounding the internal coils within tissue at a depth of 6 mm. Figure 10a shows that the total near fields on the azimuthal plane (θ = 0°, θ = 90°) are approximately −88 dB and −38 dB, respectively, and the magnitude of the electric field surrounding the coil is omnidirectional at all angles except for some specific angles. Figure 10b shows that the total near fields on the elevation plane for both (Φ = 0°, Φ = 90°) are approximately −38 dB. The magnitude of the electric field surrounding the coil is omnidirectional and there is a drop in the magnitude for some specific angles of up to −68 dB.

### 7.3. Specific Absorption Rate (SAR) Effects and Power Loss

**Figure 11.**
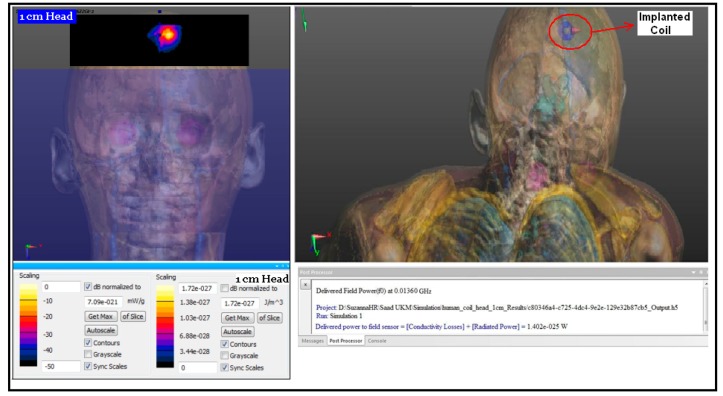
The SAR and power loss in the human body at 13.56 MHz frequency, at a depth of 10 mm.

One of the important situations of transferring signals via inductive coupling is when the tissue is facing an electromagnetic field. Such a tissue will derive the power to be dissipated inside the tissue itself, therefore the temperature will be increased around the tissue. As a result, the tissues that surround the coil may be damaged. However, power dissipation and SAR should be within the accepted range. In our design, the coils are separated by biological tissue. Thus, the SAR and power losses of the proposed coils operating at 13.56 MHz would not be damaging to the tissue. To prove that, the implanted coil was inserted at the top of the head in the space between the skull and brain at a depth of less than 1 cm. Figure 11 shows the SAR level and the power loss within the tissue where the SAR is equal 7.09 × e^−021^ mW/g, and the power loss is 1.402 × e^−025^ W. The results above show that the SAR was much less than the standard for 1 g and is negligible for 10 g. The power loss effect is negligible. Both results showed that the proposed implanted coil has a negligible SAR and a power loss effect.

## 8. Conclusions

In this study, a theoretical analysis for an inductive coupling link is optimized by using the reflected impedance method to achieve a power transmission efficiency of up to 80%. The inductive link based on a spiral circular (pancake) design was developed and tested in air and in two types of the biological human tissue (dry and wet skin) having a thickness of 6 mm, at a frequency of 13.56 MHz. The designed coils can be used for implanted micrssystem applications, where the maximum depth is 6 mm as given in the literature review. The coil values and geometries are given in Table 1. The results showed that the maximum gain surrounding the external coil in the air and on tissue is −3 dB and it is almost constant and has an omnidirectional pattern, therefore, the external coil performance in tissue is approximately 98% than it air. The internal coil performance within tissue is approximately 50% that in air. The SAR is extremely small and lower than the standard level, thus, the tissue is not heated and cannot be damaged, and power loss within the tissue is very small and can be ignored. The implant coil dimensions are small and it performed well, making it highly suitable for implanted microsystem applications.

## Figures and Tables

**Table 1.** The values and parameters of the external and internal parts.

Quantity	Symbol	External Coil	Internal Coil
Inductance	L	4.92 μH	1 μH
Series resistance	R	2.2 Ω	1.6 Ω
Capacitance	C	33.11 pf	33.11 pf
Quality Factor	Q	190	53
Outer diameter	d_out_	56 mm	11.6 mm
Inner diameter	d_in_	10 mm	0.5 mm
Number of turns	N	30	8
Inductor Width	w	0.5 mm	0.3 mm
Turn spacing	S	0.3 mm	0.1 mm
Fill factor	φ	0.69	0.39
PCB substrate	-	60 mm × 70 mm	12 mm × 15 mm
Link operating frequency	f	13.56 MHz	
Secondary nominal loading	R_load_	300 Ω	
Optimum p/Am resistance	R_P_	41.89 Ω	
Coupling Coefficients	K	0.087	
Mutual inductance	M	0.95 μH	
Coil relative distance within tissue	X	6 mm	
